# Supplementary material for: Squatting biomechanics following physiotherapist-led care or hip arthroscopy for femoroacetabular impingement syndrome: a secondary analysis from a randomised controlled trial
Source: PeerJ. 2024 Jun 24;12:e17567. doi: 10.7717/peerj.17567 (PMC11210460; doi:10.7717/peerj.17567)
Supplement: Supplemental Information 2 — Group data are mean (standard deviation) unless stated otherwise. Change data are mean difference (95% confidence interval). PHT = Personalised Hip Therapy; ARTH = arthroscopy. ⁂data collected at baseline only. ‡Data are median (range). *significant difference at follow-up compared to baseline; †significant difference in change between groups. HOOS = hip disability and osteoarthritis outcome score; iHOT-33 = International Hip Outcome Tool; UCLA = University of California Los Angeles. iHOT-33 and HOOS: 0 = extreme hip pain and impaired function, 100 = no hip pain or impaired function. UCLA activity score: 1 = wholly inactive, 10 = regular participation in high impact sports. [file peerj-12-17567-s002.docx]

**Table S1: Hip morphological and patient-reported measures at baseline and follow-up, and differences within and between groups.**

Group data are mean (standard deviation) unless stated otherwise. Change data are mean difference (95% confidence interval).

|  | **Personalised Hip Therapy (n=17)** | | | **Arthroscopy (n=19)** | | | **Difference in change** |
| --- | --- | --- | --- | --- | --- | --- | --- |
|  | **Mean baseline (SD)** | **Mean follow-up (SD)** | **Mean difference (95% CI)** | **Mean baseline (SD)** | **Mean follow-up (SD)** | **Mean difference (95% CI)** | **Mean difference (95% CI)** |
| *Hip morphology* | | | | | | | |
| Morphological classification, cam:pincer:combined | 14:2:1 |  |  | 13:2:4 |  |  |  |
| Alpha angle (°) | 73.2 (15.9) | 71.0 (14.6) | -2.2 (-12.9,8.5) | 70.0 (8.1) | 53.5 (9.8) | -16.5 (-22.5,-10.6)^*^ | -15.2 (-20.8,-9.5)^†^ |
| Lateral center edge angle (°) | 35 (7.8) | 33.6 (6.5) | -1.4 (-6.4,3.6) | 36.8 (5.3) | 35.4 (6.1) | -1.4 (-5.2,2.4) | -0.3 (-3.4,2.9) |
| *Patient-reported measures* | | | | | | | |
| iHOT-33 | 43.2 (16.6) | 67.5 (23.7) | 24.4 (10.1,38.7)^*^ | 46.9 (17.4) | 75.7 (21.6) | 28.8 (15.9,41.7)^*^ | 4.4 (-11.4,20.3) |
| UCLA activity score^⁂‡^ | 7.3 (2.8) |  |  | 7.5 (2.4) |  |  |  |
| HOOS (pain) | 63.3 (14.1) | 80.9 (13.9) | 17.6 (7.9,27.4)^*^ | 59.1 (18.6) | 86.5 (13.7) | 27.5 (16.7,38.2)^*^ | 9.4 (-4.8,23.6) |
| HOOS (symptoms) | 54.4 (21.2) | 70.7 (18.7) | 16.3 (2.4,30.3) | 50.8 (14.8) | 76.1 (16.0) | 25.3 (15.2,35.5)^*^ | 11.8 (-5.6,29.2) |
| HOOS (activities of daily living) | 75.0 (16.3) | 90.0 (10.4) | 15.0 (5.5,24.6)^*^ | 70.4 (18.9) | 92.0 (11.7) | 21.6 (11.3,32.0)^*^ | 8.3 (-3.1,19.6) |
| HOOS (sport and recreation) | 50.4 (24.8) | 75.4 (19.2) | 25.1 (9.5,40.6)^*^ | 52.0 (22.1) | 80.6 (13.7) | 28.6 (16.5,40.7)^*^ | 3.1 (-14.7,20.9) |
| HOOS (quality of life) | 34.8 (16.5) | 54.5 (23.9) | 19.7 (5.3,34.1)^*^ | 35.2 (16.8) | 65.3 (22.6) | 30.1 (17.0,43.2)^*^ | 8.8 (-7.3,24.9) |
| PHT = Personalised Hip Therapy; ARTH = arthroscopy. ^⁂^data collected at baseline only. ^‡^Data are median (range). ^*^significant difference at follow-up compared to baseline; ^†^significant difference in change between groups. HOOS = hip disability and osteoarthritis outcome score; iHOT-33 = International Hip Outcome Tool; UCLA = University of California Los Angeles. iHOT-33 and HOOS: 0 = extreme hip pain and impaired function, 100 = no hip pain or impaired function. UCLA activity score: 1 = wholly inactive, 10 = regular participation in high impact sports. | | | | | | | |
