# Supplementary material for: Squatting biomechanics following physiotherapist-led care or hip arthroscopy for femoroacetabular impingement syndrome: a secondary analysis from a randomised controlled trial
Source: PeerJ. 2024 Jun 24;12:e17567. doi: 10.7717/peerj.17567 (PMC11210460; doi:10.7717/peerj.17567)
Supplement: Supplemental Information 3 — Differences calculated as mean subsample minus mean trial samples at baseline and 12-months follow-up. aInternational Hip Outcome Tool (iHOT-33) and hip disability and osteoarthritis outcome score (HOOS): 0 = extreme hip pain and impaired function, 100 = no hip pain or impaired function. bCollected at baseline only. cUniversity of California Los Angeles (UCLA) activity score: 1 = wholly inactive, 10 = regular participation in high impact sports. [file peerj-12-17567-s003.docx]

**Table S2. Differences in demographic and clinical characteristics between subsample Personalised Hip Therapy and arthroscopy groups and trial sample.**

Differences calculated as mean subsample minus mean trial samples at baseline and 12-months follow-up.

|  | **Personalised Hip Therapy** | | **Arthroscopy** | |
| --- | --- | --- | --- | --- |
|  | **Mean difference, subsample (n=17) minus trial (n=50)** | | **Mean difference, subsample (n=19) minus trial (n=49)** | |
|  | **Baseline** | **Follow-up** | **Baseline** | **Follow-up** |
| Female, % | -7 |  | 10.6 |  |
| Age (years) | -0.3 |  | 1.0 |  |
| *Hip morphology* | | | | |
| Morphological classification, % cam:pincer:combined | 18.4:-6.2:-12.1 |  | 7.2:-8.0:0.6 |  |
| Max alpha angle (°) | 2.6 | 1.8 | -0.2 | -9.2 |
| Lateral centre edge angle (°) | -2.6 | -2.3 | -1.8 | -2.6 |
| *Patient-reported measures* | | | | |
| iHOT-33^a^ | 2.8 | 10.7 | 3.4 | 2.8 |
| UCLA activity score^b,c^ | -0.2 |  | 0.5 |  |
| HOOS (pain)^a^ | 5.9 | 9.6 | -0.3 | 2.6 |
| HOOS (symptoms)^a^ | 3.7 | 6.9 | 1.1 | 2.3 |
| HOOS (activities of daily living)^a^ | 9.1 | 13.4 | 1.2 | 2.3 |
| HOOS (sport and recreation)^a^ | -0.3 | 12.9 | 4.1 | 5.4 |
| HOOS (quality of life)^a^ | 4.7 | 8.5 | 1.6 | 2.6 |
| ^a^International Hip Outcome Tool (iHOT-33) and hip disability and osteoarthritis outcome score (HOOS): 0 = extreme hip pain and impaired function, 100 = no hip pain or impaired function.  ^b^Collected at baseline only.  ^c^University of California Los Angeles (UCLA) activity score: 1 = wholly inactive, 10 = regular participation in high impact sports. | | | | |
