# Supplementary figures and images for: Squatting biomechanics following physiotherapist-led care or hip arthroscopy for femoroacetabular impingement syndrome: a secondary analysis from a randomised controlled trial
Source: PeerJ. 2024 Jun 24;12:e17567. doi: 10.7717/peerj.17567 (PMC11210460; doi:10.7717/peerj.17567)

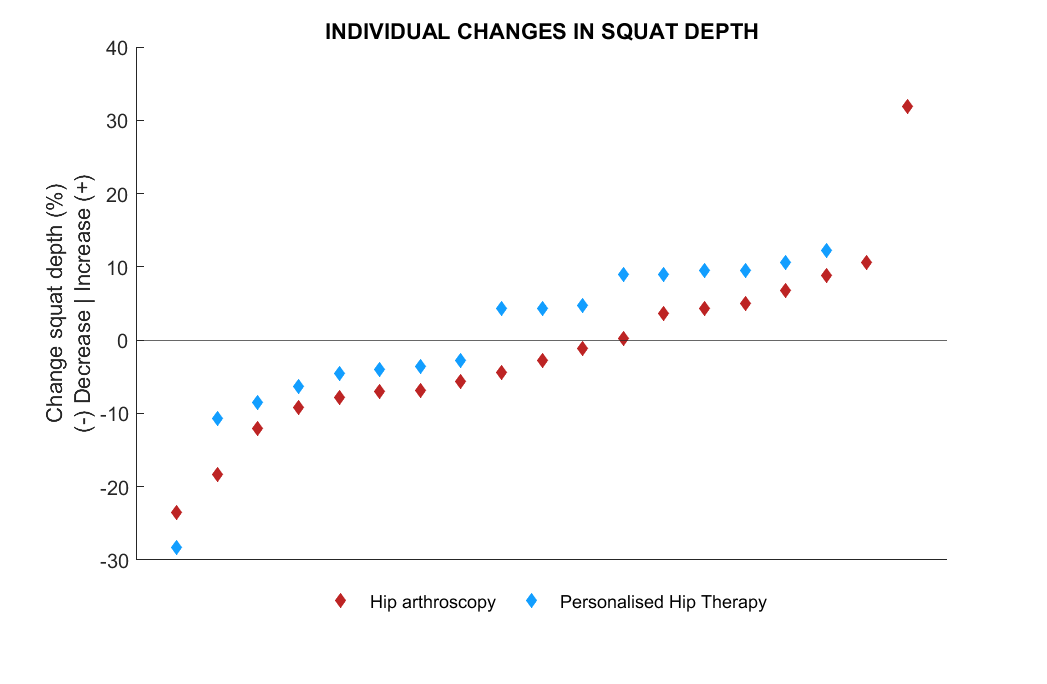

Supplement: Supplemental Information 4 — Individual participant changes (follow-up minus baseline) in maximum squat depth relative to starting height (change in vertical position of the midpoint of the two sacral markers, expressed as a percentage of limb length, %). The number of participants who decreased or increased squat depth at follow-up relative to baseline in the Personalised Hip Therapy (blue) and hip arthroscopy (red) groups were similar. [file peerj-12-17567-s004.png]
