# Supplementary material for: Squatting biomechanics following physiotherapist-led care or hip arthroscopy for femoroacetabular impingement syndrome: a secondary analysis from a randomised controlled trial
Source: PeerJ. 2024 Jun 24;12:e17567. doi: 10.7717/peerj.17567 (PMC11210460; doi:10.7717/peerj.17567)
Supplement: Supplemental Information 8 — Source: Murphy, N.J., Eyles, J., Bennell, K.L. et al. Protocol for a multi-centre randomised controlled trial comparing arthroscopic hip surgery to physiotherapy-led care for femoroacetabular impingement (FAI): the Australian FASHIoN trial. BMC Musculoskelet Disord 18, 406 (2017). https://doi.org/10.1186/s12891-017-1767-y [file peerj-12-17567-s008.pdf]

Murphy et al. BMC Musculoskeletal Disorders (2017) 18:406
DOI 10.1186/s12891-017-1767-y

BMC Musculoskeletal Disorders

STUDY PROTOCOL

Open Access

Protocol for a multi-centre randomised controlled trial comparing arthroscopic hip surgery to physiotherapy-led care for femoroacetabular impingement (FAI): the Australian FASHIoN trial

Nicholas J. Murphy[1,2†], Jillian Eyles[1,2†], Kim L. Bennell[3], Megan Bohensky[4], Alexander Burns[5], Fraser M. Callaghan[6,7], Edward Dickenson[8], Camdon Fary[9,10], Stuart M. Grieve[6,7], Damian R. Griffin[8], Michelle Hall[3], Rachel Hobson[8], Young Jo Kim[11], James M. Linklater[12], David G. Lloyd[13,14], Robert Molnar[15], Rachel L. O'Connell[2,16], John O'Donnell[17,18], Michael O'Sullivan[19], Sunny Randhawa[20], Stephan Reichenbach[21,22], David J. Saxby[13,14], Parminder Singh[17,23], Libby Spiers[3], Phong Tran[9,10], Tim V. Wrigley[3] and David J. Hunter[1,2*]

Abstract

Background: Femoroacetabular impingement syndrome (FAI), a hip disorder affecting active young adults, is believed to be a leading cause of hip osteoarthritis (OA). Current management approaches for FAI include arthroscopic hip surgery and physiotherapy-led non-surgical care; however, there is a paucity of clinical trial evidence comparing these approaches. In particular, it is unknown whether these management approaches modify the future risk of developing hip OA. The primary objective of this randomised controlled trial is to determine if participants with FAI who undergo hip arthroscopy have greater improvements in hip cartilage health, as demonstrated by changes in delayed gadolinium-enhanced magnetic resonance imaging (MRI) of cartilage (dGEMRIC) index between baseline and 12 months, compared to those who undergo physiotherapy-led non-surgical management.
(Continued on next page)

* Correspondence: david.hunter@sydney.edu.au
†Equal contributors
[1]Kolling Institute of Medical Research, Institute of Bone and Joint Research, University of Sydney, Camperdown, Australia
[2]Department of Rheumatology, Royal North Shore Hospital, St Leonards, Australia
Full list of author information is available at the end of the article

BioMed Central

© The Author(s). 2017 **Open Access** This article is distributed under the terms of the Creative Commons Attribution 4.0 International License (http://creativecommons.org/licenses/by/4.0/), which permits unrestricted use, distribution, and reproduction in any medium, provided you give appropriate credit to the original author(s) and the source, provide a link to the Creative Commons license, and indicate if changes were made. The Creative Commons Public Domain Dedication waiver (http://creativecommons.org/publicdomain/zero/1.0/) applies to the data made available in this article, unless otherwise stated.

(Continued from previous page)

Methods: This is a pragmatic, multi-centre, two-arm superiority randomised controlled trial comparing hip arthroscopy to physiotherapy-led management for FAI. A total of 140 participants with FAI will be recruited from the clinics of participating orthopaedic surgeons, and randomly allocated to receive either surgery or physiotherapy-led non-surgical care. The surgical intervention involves arthroscopic FAI surgery from one of eight orthopaedic surgeons specialising in this field, located in three different Australian cities. The physiotherapy-led non-surgical management is an individualised physiotherapy program, named Personalised Hip Therapy (PHT), developed by a panel to represent the best non-operative care for FAI. It entails at least six individual physiotherapy sessions over 12 weeks, and up to ten sessions over six months, provided by experienced musculoskeletal physiotherapists trained to deliver the PHT program. The primary outcome measure is the change in dGEMRIC score of a ROI containing both acetabular and femoral head cartilages at the chondrolabral transitional zone of the mid-sagittal plane between baseline and 12 months. Secondary outcomes include patient-reported outcomes and several structural and biomechanical measures relevant to the pathogenesis of FAI and development of hip OA. Interventions will be compared by intention-to-treat analysis.

Discussion: The findings will help determine whether hip arthroscopy or an individualised physiotherapy program is superior for the management of FAI, including for the prevention of hip OA.

Trial registration: Australia New Zealand Clinical Trials Registry reference: ACTRN12615001177549. Trial registered 2/11/2015 (retrospectively registered).

Keywords: Arthroscopy, dGEMRIC, Femoroacetabular impingement syndrome, Fai, Hip, Orthopaedic, Osteoarthritis, Physiotherapy, Surgery

Background

FAI syndrome (FAI) is a motion-related clinical disorder of the hip with a triad of symptoms, clinical signs, and imaging findings [1]. It represents symptomatic premature contact between the proximal femur and acetabulum. Two morphologic patterns of FAI have been described: cam type, where the femoral head is aspheric due to a thickened femoral head-neck junction; and pincer type, where the acetabular rim extends beyond its normal depth to over-cover the femoral head [2]. Both cam and pincer type morphology are associated with the repetitive abutment of the proximal femur against the acetabular rim, applying shear forces to the acetabular labrum and/or cartilage, which is believed to lead to hip osteoarthritis (OA) [3–5]. The evidence linking FAI to the development of hip OA is rapidly expanding, such that FAI is now believed to be a precursor to what was previously described as primary or idiopathic hip OA [2, 6–11].

Hip OA is associated with reduced quality of life and high healthcare costs [12]. Effective treatments for FAI may reduce the risk of hip OA as well as alleviating symptoms of the syndrome itself. Current popular management approaches for FAI include surgery, most commonly in the form of hip arthroscopy, and physiotherapy-led non-surgical care. There is a notable lack of high-quality research comparing outcomes between these two management approaches [13, 14]. In particular, it is unknown whether either of these approaches modifies the risk of future hip OA development.

Ganz and colleagues first described the pathomechanism of FAI, as well as an open approach to surgically correct the associated bony abnormalities, at the turn of the twenty-first century [2, 4, 5]. Over the last decade, arthroscopic hip surgery for FAI has been performed at a growing rate worldwide [15, 16]. Arthroscopic FAI surgery involves resection of the cam and/or pincer morphology, and usually involves surgical repair of concomitant FAI-associated soft tissue pathology, such as acetabular labral tears and chondral defects. Case series have reported positive outcomes from hip arthroscopy for FAI [17–20], however, no randomised controlled trials (RCTs) have yet been completed that compare hip arthroscopy to other interventions or to sham surgery [13]. As such, the effectiveness of hip arthroscopy for FAI remains unknown.

Non-surgical management of FAI has encompassed various methods, the mainstay of which has been physiotherapy-led exercise rehabilitation, oftentimes accompanied by anti-inflammatories or corticosteroid injections [14, 21]. Central aspects of physiotherapy-led management typically include a progressive physiotherapist supervised rehabilitation exercise program, education about FAI, and pain relief, particularly anti-inflammatory medications and sometimes intra-articular corticosteroid injections [21]. The focus of rehabilitation exercise programs is the restoration of hip muscle function and strength, to improve control of the femoral head, thereby reducing hip impingement in positions of substantial hip flexion, adduction and internal rotation [22–24]. As with surgery, there is a paucity of RCT evidence to determine whether physiotherapy-led non-surgical care is efficacious in treating FAI, and how its efficacy compares to other management approaches [14].

Rationale

There is a need for RCTs comparing arthroscopic hip surgery to physiotherapy-led non-surgical care for the treatment of FAI [13]. Given the probable causative role that FAI plays in hip OA, an important criterion by which each treatment must be evaluated is its effect on risk of future hip OA. A major problem encountered in investigating such an outcome is that the onset of OA, and thus a meaningful comparison between treatment groups, takes several decades to occur. A different approach is needed to direct current clinical practice. This RCT will measure several structural and biomechanical outcomes relevant to the pathogenesis of hip OA, with the aim of determining whether hip arthroscopy and physiotherapy-led non-surgical care differ in their effect on risk of future hip OA. This RCT is being conducted in collaboration with the UK FASHIoN trial (Trial Registration number: ISRCTN64081839) [25], which shares identical physiotherapy and surgical protocols to the Australian FASHIoN trial. Whereas the UK FASHIoN trial is focused on comparing patient-reported outcomes between the two interventions, the Australian FASHIoN trial is primarily investigating mechanistic outcomes.

Objectives of the Australian FASHIoN trial

Primary objective

To compare 12-month changes in hip cartilage health between treatment groups, as demonstrated by changes in the average dGEMRIC score for a region of interest (ROI) including both acetabular and femoral head cartilage cartilages at the chondrolabral transitional zone.

Secondary objectives

- Compare 12-month change in hip cartilage health between treatment groups, as demonstrated by changes in the dGEMRIC scores of separate acetabular and femoral head cartilage ROIs at the chondrolabral transitional zone.
- Compare 12-month change in hip MRI and plain X-ray features between treatment groups, using the Hip Osteoarthritis MRI Scoring System (HOAMS) and Hip2Norm software, respectively.
- Compare 12-month change in hip joint three-dimensional stresses and strains during walking and various functional tasks between treatment groups.
- Determine if changes in hip joint structure after 12 months are related to symptomatic improvements.
- Compare 12-month change in hip muscle activation patterns and co-contraction patterns between treatment groups.
- Compare 12-month change in hip joint motion and moments in gait between treatment groups.
- Compare 12-month change in hip isometric muscle strength between treatment groups.
- Compare 12-month change in hip joint contact forces between treatment groups.

The objectives of the UK FASHIoN trial [25] are secondary objectives of our trial, and include:

- Compare changes in patient-reported hip-specific quality of life after 12 months, as measured by the international Hip Outcome Tool-33 (iHOT-33).
- Compare changes between treatment groups in general health status and health-related quality of life after 12 months, as measured by the Short Form-12 (SF-12).
- Compare differences between treatment groups in patient satisfaction with treatment and outcome after 12 months.
- Compare differences between treatment groups in the number and severity of adverse events after treatment after 12 months.
- Compare differences between treatment groups in the need for further surgical procedures up to three years.
- Compare the cost-effectiveness of hip arthroscopy for FAI with non-surgical care, within the trial, and estimated for a patient's lifetime.
- Measure fidelity of delivery of interventions.

Methods/design

Trial design

This trial will be conducted in compliance with the Australian National Health and Medical Research Council (NHMRC) National Statement on Ethical Conduct in Human Research (2007), the Note for Guidance on Good Clinical Practice (CPMP/ICH-135/95), and the conditions of the ethics approval granted by St Vincent's Hospital Human Research Ethics Committee (HREC/14/SVH/343). Results for this trial will be reported in accordance with the CONSORT statement.

The protocol for Australian FASHIoN is similar to that for the parallel UK FASHIoN trial [25], and benefits from the feasibility study performed for the UK trial [26, 27] and from collaboration between the Australian and UK research groups. Inclusion and exclusion criteria, and intervention specifications are identical. Patient-reported outcomes and timing of those outcome assessments are the same. The key differences are the mechanistic structural and biomechanical outcomes investigated in the Australian FASHIoN trial.

The Australian FASHIoN trial is a pragmatic, assessor- and statistician-blinded, two-arm superiority RCT with 1:1 allocation ratio. 140 participants will be recruited from the private and/or public clinics of eight orthopaedic

surgeons, spread across three regions in Australia. Recruitment and/or arthroscopic surgery will be carried out at the following hospital sites; in New South Wales (NSW): Mater Hospital, Norwest Hospital, Sydney Adventist Hospital, St George Private Hospital, St Luke's Hospital, Sutherland Hospital; in Victoria (VIC): Maroondah Hospital, St Vincent's Private Hospital (East Melbourne), Western Health; and in the Australian Capital Territory (ACT): Canberra Hospital, Calvary Hospital. The PHT program will be administered at various private physiotherapy clinics located throughout NSW, VIC and ACT.

PHT will commence after treatment allocation and continue for a maximum of six months, with six compulsory physiotherapy sessions within the first 12 weeks and up to ten sessions over the first six months if needed by the participant. Arthroscopic hip surgery will occur as soon as practicable and no more than 18 weeks after treatment allocation. Post-operative rehabilitation will not be standardised, and will occur as per each participating orthopaedic surgeon's usual rehabilitation protocol. The primary outcome will be measured 12 months post-randomisation. For further details, see Trial Design flow chart (see Fig. 1).

Eligibility criteria

Participants will be eligible for participation in the trial if they meet all of the following inclusion criteria and none of the exclusion criteria.

Inclusion criteria

- Age > 16 years;
- Symptoms of hip pain that may include clicking, catching, and/or giving way;
- Radiological signs of FAI, defined as:
  - Alpha angle >55° for cam morphology [28]; and/or
  - Lateral centre edge angle >40° or other radiographic signs of pincer morphology, such as positive cross-over sign [29].

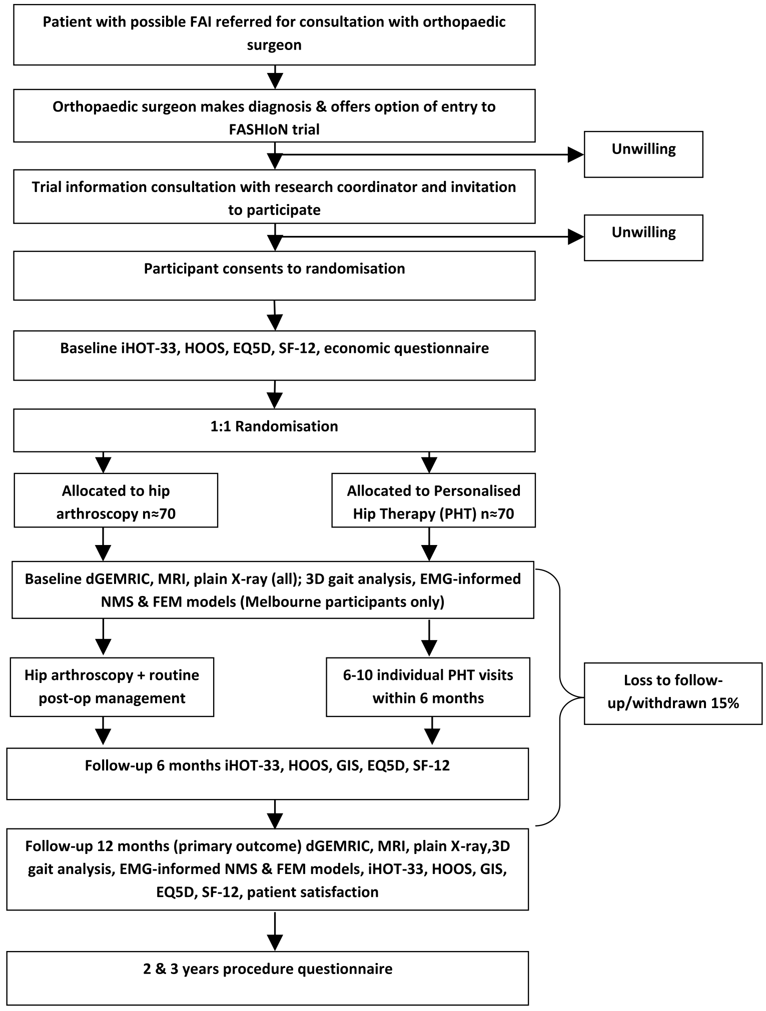

Fig. 1 Trial Design Flow Chart

- Treating surgeon believes the patient would benefit from arthroscopic FAI surgery;
- Willing and able to give written informed consent and participate fully in the interventions and follow-up procedures.

Exclusion criteria

- Evidence of pre-existing hip OA in the hip being considered for treatment in the study, defined as Tonnis grade > 1 or more than 2 mm loss of superior joint space width on anteroposterior pelvic radiograph [20, 30];
- Previous significant hip pathology such as Perthes' disease, slipped upper femoral epiphysis or avascular necrosis;
- Previous hip injury such as acetabular fracture, hip dislocation or femoral neck fracture;
- Previous shape changing surgery (open or arthroscopic) to the hip being considered for treatment;
- Inability to have MRI with contrast (e.g. due to renal impairment, pregnancy, or breast-feeding).

Recruitment

Eligible patients will be recruited via the private practice and/or public hospital clinics of eight orthopaedic surgeons, located in three different Australian cities, specialising in arthroscopic hip surgery. Orthopaedic surgeons will assess patients as usual, taking a history, performing a physical examination, and obtaining further investigations as appropriate. Patients in whom a diagnosis of FAI is made, and who meet the eligibility criteria, will be offered a trial information consultation with a trained clinical researcher and be invited to provide their informed written consent to join the FASHIoN study. Patients with bilateral FAI will be asked to nominate their most symptomatic hip satisfying the eligibility criteria for inclusion in the study.

Randomisation

Randomisation to either arthroscopic hip surgery or non-surgical physiotherapy-led care will occur in a 1:1 ratio using a computer-generated minimisation sequence (adaptive stratified sampling) with study site and type of FAI (cam, pincer or mixed FAI) as factors. Allocation concealment will be preserved by having randomisation codes held by an external biostatistician. At randomisation, participants will be given a study ID that will be used on all trial documentation.

Blinding

Neither participants, nor treating surgeons/physiotherapists, can be blinded to treatment allocation in this study. The treating surgeons and physiotherapists will take no part in outcome assessment for the trial. Imaging and biomechanical analyses will be performed in a blinded fashion. The patient reported outcome data will be collected via online surveys and postal questionnaires, which will be entered onto the central trial database by a research assistant blinded to treatment allocation.

Interventions

Arthroscopic hip surgery

Arthroscopic surgery will be standardised and performed by one of eight orthopaedic surgeons experienced in arthroscopic hip surgery for FAI. Participants will access surgery through either the public healthcare system, with no out-of-pocket cost, or through the private healthcare system, typically associated with additional out-of-pocket costs. After giving written informed consent for the procedure, patients will undergo routine preoperative care, including an anaesthetic consultation to assess surgical fitness. Surgery will be performed under general anaesthesia in either a lateral or supine position. Arthroscopic portals will be established in the central and peripheral compartment under radiographic guidance, according to each surgeon's usual practice. Shape abnormalities and consequent labral and cartilage pathologies will be treated. Bony resection at the acetabular rim and the head-neck junction will be assessed by intraoperative image intensifier radiograph and/or satisfactory impingement free range of movement of the hip. Osteo-integrative anchors will be used during the surgical procedure to avoid issues with post-operative MRI quality and dGEMRIC accuracy.

Patients will be discharged from the hospital when they can walk safely with crutches (usually within 24 h). A protocol for post-operative rehabilitation will not be specified, although all patients will be instructed to follow the usual post-operative rehabilitation protocol recommended by their surgeon. Physiotherapists providing post-operative rehabilitation care will be distinct from those providing the physiotherapy-led non-surgical care in this trial to avoid contamination between groups.

To assess the fidelity of the treatment received to the prescribed surgical protocol, an international panel of surgeons specialised in hip arthroscopy will review a random sample of the participants treated by each of the study surgeons. Operation notes, intraoperative images, and postoperative MRI scans will be used to evaluate the adequacy of the surgical intervention.

Physiotherapy-led non-surgical care

The physiotherapy-led non-surgical care provided to participants through this trial is called Personalised Hip Therapy (PHT) [21]. It was designed to represent a consensus on the best non-surgical care for FAI by an international panel of physiotherapists, physicians, and surgeons. PHT

program will be provided at no cost to participants and will be delivered by experienced musculoskeletal physiotherapists trained in its delivery. Physiotherapists delivering the PHT program will take part in a one-day training course explaining the rationale for the FASHIoN trial and the PHT program, and instruction on how to record data related to fidelity of the intervention delivery. Participants will undergo a minimum of six PHT sessions during the first 12 weeks of the study, commencing as soon after randomisation as is practicable. If needed, participants may have additional PHT sessions between 12 weeks and six months, with a maximum of ten sessions provided by the study. Further physiotherapy treatment beyond the ten sessions provided by the PHT physiotherapist will not form part of the PHT protocol and will be recorded as a co-intervention.

The PHT program encompasses a multi-faceted approach, beginning with an assessment of the patient's pain, function and hip range of motion. The core aspects of the program include (i) an individualised and progressive exercise programme supervised by a physiotherapist, (ii) education as to the condition and its management, and (iii) advice regarding pain relief which may include referral to the participants' General Practitioner, or if necessary referral for an ultrasound-guided intra-articular steroid injection to enable participants to engage in the exercise programme where pain would otherwise prevent them from doing so. The PHT program represents a structured and individualised approach to FAI management. Physiotherapists will be provided with a set of recommended exercises as part of the program, from which they will prescribe appropriate exercises for each participant's stage of rehabilitation. Participants will be given a logbook to record the exercises they complete at home. Data from the logbook will not be collected by researchers, but will be used to enhance physiotherapist-patient communication and facilitate the development of strategies to maximise patient adherence to the prescribed exercises.

The key features of the exercise programme are individualisation, progression and supervision; thus, evidence of these features will be sought from individual participant PHT case report forms (CRFs). A randomly selected sample of CRFs for participants treated by each of the PHT physiotherapists will be assessed members of the panel that developed the PHT protocol to assess treatment fidelity.

Outcomes

The primary outcome measure for analysis will be the 12-month change in the average T_1 relaxation time, assessed with dGEMRIC, for a ROI comprising both acetabular and femoral head cartilages at the chondrolabral transitional zone of the mid-sagittal plane. The 12-month change in separate acetabular and femoral head cartilage ROIs at the chondrolabral transitional zone will be analysed as a secondary outcome. In addition, the 12-month change in standardised dGEMRIC z-scores for the acetabular and femoral head cartilage ROIs at the chondrolabral junction will be analysed, using the central femoral cartilage ROI as an internal control [31, 32]. The dGEMRIC technique has been proven reliable for quantification and detection of changes in the glycosaminoglycan (GAG) content of the hip joint cartilage [33–36]. Since the loss of GAG in cartilage is an early OA-related change [37], dGEMRIC enables the likelihood of future OA development to be compared between treatment methods after a relatively short period. Further detail regarding the dGEMRIC outcome measurements and analyses is included below.

Other secondary outcomes will include hip joint structural change between baseline and 12 months as demonstrated by the semi-quantitative whole Hip OA MRI Score (HOAMS), and by plain radiographic and MRI measures of alpha angle, acetabular depth, femoral and acetabular version; change between baseline and 12 months in hip joint biomechanics during gait and functional tasks as measured by external hip joint motion and moments, muscle activation and co-contraction patterns, net hip joint contact forces in localised regions of cartilage, and regional cartilage stresses and strains; cost-effectiveness of the interventions as measured by the modified World Health Organisation Health and Work Performance Questionnaire (WHO HPQ) [38]; changes in participant symptoms and quality of life as measured by the international Hip Outcome Tool-33 (iHOT-33) [39], the Hip disability and Osteoarthritis Outcome Score (HOOS) [40–42], EQ-5D [43], and the 12-Item Short Form Health Survey (SF-12) [44]; patient perceived overall improvement following intervention measured using a Global Improvement Scale (GIS); and patient satisfaction with care and treatment results measured on a five-point Likert scale. Procedures for measurement and analysis of each of the secondary outcomes is described in greater detail below. The table of data collection and time points (see Table 1) summarises the timeline for each of the study procedures.

MRI

All participants will undergo an MRI scan at baseline and 12 months, performed on one of three 3 T MRI scanners using a phased array coil on a Siemens Prisma (Melbourne), Siemens Skyra (Sydney), or Phillips Ingenia (Canberra). Participants will be scanned on the same scanner at baseline and 12 months. Participants will receive an intravenous injection of the contrast agent; 0.2 mmol/kg bodyweight of Dotarem (Gd-DOTA; Guerbet, Cedex, France) or Magnevist (Gd-DTPA; Berlex

Table 1 Table of data collection and time points

Time points:	Baseline	6 months	12 months	24 & 36 months
Data collection:	Demographic information<br>Physical activity (modified UCLA Activity Scale)<br>MRI scan<br>dGEMRIC scan<br>Plain radiography<br>3D gait analysis<br>EMG-informed NMS and FEM models<br>iHOT-33<br>HOOS<br>SF-12<br>EQ-5D<br>WHO HPQ (modified)	iHOT-33<br>HOOS<br>GIS<br>SF-12<br>EQ-5D<br>WHO HPQ (modified)	MRI scan<br>dGEMRIC scan<br>Plain radiography<br>3D gait analysis<br>EMG-informed NMS and FEM models,<br>iHOT-33<br>HOOS<br>GIS<br>SF-12<br>EQ-5D<br>Patient satisfaction<br>Resource utilization<br>Adverse events<br>WHO HPQ (modified)	Further procedures questionnaire

dGEMRIC = delayed gadolinium-enhanced magnetic resonance imaging of cartilage, EMG-informed NMS and FEM models = electromyography-informed neuromusculoskeletal and finite element models, iHOT-33 = international Hip Outcome Tool-33, HOOS = Hip disability and Osteoarthritis Outcome Score, SF-12 = Short-Form 12, European Quality of life-5 dimensions, WHO HPQ = World Health Organisation Health and Work Performance Questionnaire, GIS = Global Improvement Score

Labs, Wayne, NJ). Following injection of contrast agent, participants will walk for 15 min, after which MRI scanning will occur.

The following MRI sequences, all using a surface coil, will be part of the protocol: coronal and axial fat-suppressed T_1-weighted spin-echo sequence (TR 600 ms, TE 7.9 ms, slice thickness/slice gap 3.0 mm/0.3 mm, echo train length 3, field of view (FOV) 18 × 18 cm, matrix size 256 × 256, number of signal averages 1, number of slices 24), coronal and sagittal proton density-weighted fat-suppressed fast spin-echo sequence (TR 2230 & 2770 ms, TE 29 & 36 ms, slice thickness/slice gap 3.0 mm/0.3 mm, echo train length 7 & 9, FOV 18 × 18 cm, matrix size 256 × 256, number of signal averages 2), a sagittal 3D T_2-weighted true fast imaging with steady-state precession sequence (TR 10.2 ms, TE 4.3 ms, slice thickness 0.63 mm, FOV 16 × 16 cm, matrix size 256 × 256, number of signal averages 1), an axial fat-suppressed T_1-weighted spin-echo sequence of the pelvis covering the hip joints (TR 500 ms, TE 8.9 ms, slice thickness/slice gap 3.0 mm/0.9 mm, echo train length 3, FOV 36 × 36 cm, matrix size 256 × 256, number of signal averages 1), an axial fat-suppressed T_1-weighted spin-echo sequence of the knees (TR 550 ms, TE 11 ms, slice thickness/slice gap 5.0 mm/1.5 mm, echo train length 4, FOV 32 × 32, number of signal averages 2), an axial fat suppressed T_1-weighted spin-echo sequence of the ankles at the Melbourne site only (TR 470 ms, TE 12 ms, slice thickness / slice gap 5.0/1.5 mm, echo train length 3, FOV 36 × 36 cm, matrix size 320 × 320, number of signal averages 1), and the dGEMRIC sequences (spin-echo inversion recovery with fat suppression; sagittal orientation, TR 2340 ms, TE 15 ms, slice thickness/slice gap 3.0 mm/3.0 mm, echo train length 11, FOV 16 × 16 cm, matrix size 256 × 256, number of signal averages 1; 6 IR delays at 50, 100, 200, 400, 800, and 1600 ms). Acquisition of dGEMRIC sequences will occur in the 45–60-min time window (following injection), consistent with previously performed dGEMRIC protocols. The total time for the MRI examination including patient positioning will be approximately 45 min, excluding walking and contrast injection.

MRI sequences were chosen to enable scoring with the HOAMS, a validated and reliable semi-quantitative whole hip osteoarthritis MRI scoring system [45]. Changes in HOAMS scores between baseline and 12 months will be reported. The MRI measures of FAI morphology will be reported both at baseline and 12 months, including alpha angle, acetabular version and femoral version. Alpha angle will be measured in 30-degree intervals from the 'superior' to 'anterior' locations, with the largest alpha angle and its location to be reported. Acetabular version will be measured using the T_1-weighted axial hips sequence and will be reported at 1 cm from the roof of the acetabulum and at the centre of the acetabulum. Femoral version will be measured on the T_1-weighted axial hip and knee sequences and will be defined as the difference between the angle made by the long axis of the femoral neck and the angle made by the posterior distal femoral condyles, as described previously [46].

dGEMRIC

Following MRI acquisition, dGEMRIC analysis will be carried out using a methodology closely based on that validated in previously published studies [47]. Acetabular and femoral head cartilage ROIs will be defined for three mid-sagittal plane slices at the chondrolabral transitional zone, reaching approximately 3 to 6 mm toward the acetabular fossa in each hip. Accurate positioning of the mid-sagittal plane was ensured using a three-dimensional view of the hip volume in Osirix (version 8, Geneva, Switzerland [48]; see Fig. 2). This cartilage subregion has been chosen for analysis due to its relevance in the pathogenesis of FAI [4, 49]. Care

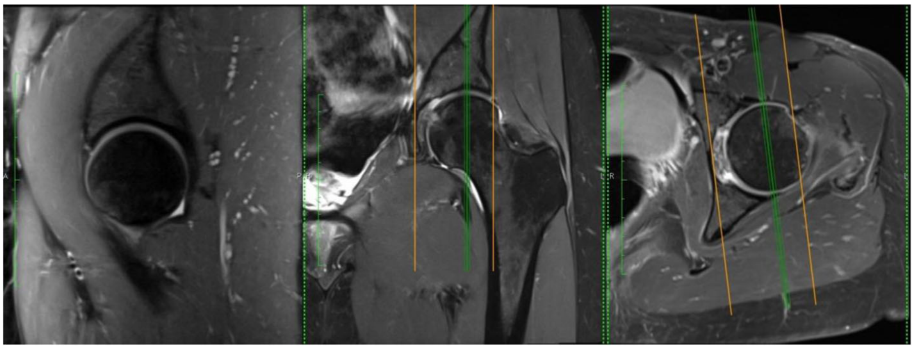

Fig. 2 Selection of mid-sagittal plane for dGEMRIC analysis. Selection of the mid-sagittal plane using OsiriX. dGEMRIC analysis will be performed on the three mid-sagittal slices

will be taken to define ROIs including only acetabular and femoral head cartilage separately, while excluding labrum and bone. The mean change in T_1 values between baseline and 12 months for these ROIs will be reported.

We will also calculate z-scores for the acetabular and femoral cartilage ROIs at the chondrolabral junction, using the standardised dGEMRIC method proposed by Lattanzi et al. [31, 32]. Standardised dGEMRIC uses the healthy central femoral head cartilage as an internal control to which other cartilage ROIs can be compared, by transforming the T_1 values (x) to standard scores (z) using $z = (x - \mu) / \sigma$, where μ and σ are the mean and standard deviation, respectively, of T1 values within the central femoral head cartilage ROI. Standardised dGEMRIC enables better comparison of local cartilage damage between different individuals as it removes the effect of individual-level factors affecting T_1 values such as age, sex, BMI, and differences in gadolinium diffusion and transport rates through cartilage [31]. Standardised dGEMRIC z-scores will be reported at baseline and 12 months for acetabular and femoral ROIs at the chondrolabral junction, with a central femoral cartilage ROI to be used as an internal control. By measuring and reporting the change in standardised dGEMRIC scores between baseline and 12 months, we will mitigate the effect that surgical trauma has been posited to have in reducing cartilage T_1 values globally for a period of time following surgery [50, 51]. Change in standardised dGEMRIC z-scores between baseline and 12 months may provide a more accurate reflection of local changes in cartilage health at the FAI-relevant chondrolabral junction.

Plain radiograph

All participants will have plain X-rays at baseline and 12 months. At baseline, a supine anterior-posterior (AP) pelvis, 45-degree modified Dunn view and false profile view X-ray will be acquired. At 12 months, a supine AP pelvis and modified Dunn view will be acquired. A detailed protocol will be provided to radiographers to ensure consistent patient and X-ray beam positioning between cases [52]. A comprehensive set of radiographic measurements applicable to FAI pathoanatomy will be measured using Hip2Norm software, which has been validated for this purpose [53]. These measures include: pelvic orientation, acetabular anteversion, acetabular depth, acetabular inclination, femoral head coverage, femoral head sphericity, joint space width, and joint congruity. These data will be reported at baseline and 12 months, enabling characterisation of the effects of surgery on the pathoanatomy of FAI.

Motion analysis EMG data collection

Participants enrolled in Melbourne will undergo motion analysis during walking, squatting, and jogging at baseline and 12 months. A 12-camera Vicon motion capture system (Vicon, Oxford Metrics, UK) will be used to acquire three-dimensional motion data of participants wearing a full-body marker set with modified lower limb 10-marker clusters at 120 Hz) [54]. Ground reaction forces will be acquired using two AMTI (Advanced Mechanical Technology Institute, Massachusetts, USA) force plates sampling at 1200 Hz. electromyograms will be acquired with a Noraxon DTS 2400 wireless telemetry system from 14 muscles of the study limb, sampling at 1200 Hz. According to surface electromyography for the non-invasive assessment of muscles (SENIAM) guidelines [55], surface electrodes will be placed on the soleus, medial and lateral gastroncnemii, hamstrings, vasti, rectus femoris, tibialis anterior, peroneals, adductors, tensor fasciae latae, gluteus maximus, and gluteus medius. Biomechanical outcome parameters will include tri-planar kinematics and kinetics of the lower-limb segments and joints, and relevant pelvis and trunk kinematics.

Calibrated EMG-informed Neuromusculoskeletal (CEINMS) [56] and Finite Element Method (FEM) models will be used to calculate stress and strain fields, as well as strain energy density, of the acetabular and

femoral cartilages to determine cartilage loading stimulus at baseline and 12 months [57]. The MRI and gait analysis data will be employed to create patient-specific lower-limb rigid body musculoskeletal models and FEM hip meshes using the Musculoskeletal Atlas Project (MAP) software [58]. The EMG-informed muscle-tendon and joint contact forces will be determined using CEINMS and then applied as force-boundary conditions to a FEM model of the hip. We will then resolve the stress and strain distributions in the hip bones and cartilages using FEBio (febio.org), which is validated, Open-source FEM software specifically designed for biomechanics applications [59].

Hip muscle strength assessment

Participants who attend the gait laboratory for motion analysis will have their hip muscle strength assessed at baseline and 12 months. A digital dynamometer force gauge (Sparker Instruments, China) will be used to assess peak isometric hip flexion, extension, adduction, and abduction strengths. For each hip strength assessment, participants will stand upright within a testing frame with the force transducer secured via a perpendicular strap around the lower thigh to the frame (see Fig. 3). The distance from the greater trochanter to the force transducer thigh attachment will be recorded as the lever arm distance in metres (m). For the study leg, participants will perform two submaximal efforts for familiarisation. Participants will then perform two maximal efforts and will be encouraged to pull as hard they can for 3–5 s with a short rest period between each of the two maximal trials. From the two maximal trials, the peak torque in Newton metres (Nm) will be determined and normalised to body mass (Nm/kg).

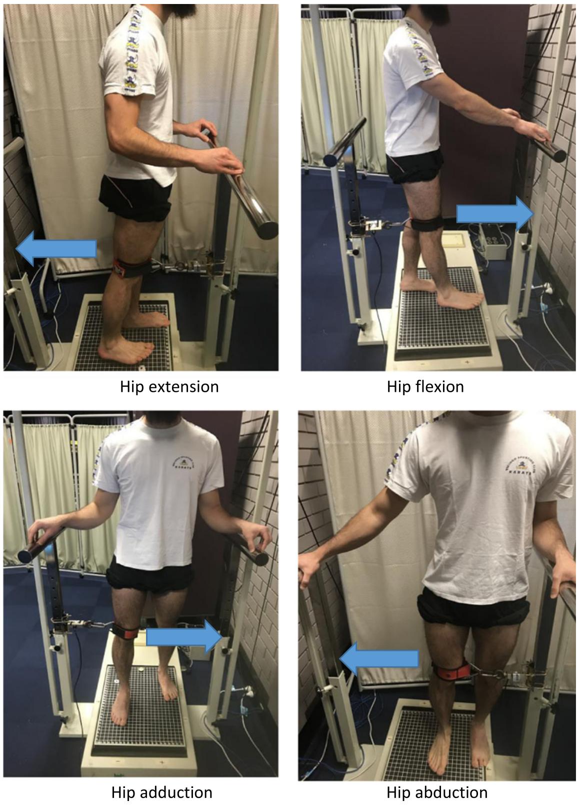

Fig. 3 Hip muscle strength assessment. For hip muscle group strength assessment, participants are instructed to stand upright, look straight ahead and use the rail for light finger-tip support. Following familiarisation participants receive strong verbal encouragement to pull as hard as possible. Two maximal efforts for each hip muscle group are recorded

Patient reported outcomes

Patient reported outcomes will be collected at baseline, 6 months, and 12 months, as described in Table 1. The modified World Health Organisation Health and Work Performance Questionnaire (WHO HPQ) [38] will be utilised to assess cost-effectiveness of the interventions. Changes in participant symptoms and quality of life will be assessed using the iHOT-33 [39], the HOOS [40–42], EQ-5D [43] and the SF-12 [44]. A Global Improvement Scale (GIS) will be used to determine patient perceived overall improvement at 6 months and 12 months following baseline. Patient satisfaction will be measured on a five-point Likert scale after 12 months, in response to the questions, *"Overall, how satisfied are you with the treatment you received?"* and *"Overall, how satisfied are you with the results of your treatment?"*.

Need for further procedures

The need for any further treatments will be recorded for participants in both arms of the study. Further treatments may include but are not limited to hip arthroscopy, open hip preservation surgery, hip replacement, or additional non-protocol physiotherapy. The need for further procedures will be ascertained by questionnaire at two years and three years.

Adverse events

The number and type of adverse events will be recorded for all participants up to 12 months. Adverse events (AE) are defined as any untoward medical occurrence in a clinical trial patient and which do not necessarily have a causal relationship with the treatment. All AEs will be listed on the appropriate Case Report Form for routine return to the researchers. Serious adverse events (SAE) are defined as any untoward and unexpected medical occurrence that: results in death; is life-threatening; requires hospitalisation or prolongation of existing inpatients hospitalisation; results in persistent or significant disability or

incapacity; is a congenital anomaly or birth defect; or any other important medical condition which, although not included in the above, may require medical or surgical intervention to prevent one of the outcomes listed. All SAEs will be reported to the ethics committee within 72 h of the investigators becoming aware of them. All participants experiencing SAEs will be followed-up as per protocol until the end of the trial.

Statistical analysis

Descriptive analysis

Data will be checked for outliers and missing values, and validated using the defined score ranges for all outcome measures. Standard statistical summaries (medians, ranges, means, standard deviations; dependent on the distribution of the outcome) will be presented for all outcome measures. Scatter plots depicting the relationship between relevant outcomes will be presented with corresponding correlation coefficients. Counts with percentages will be presented for categorical variables. Baseline data will be summarized to check comparability between treatment arms, and to highlight any systematic differences between those individuals in the study, those ineligible, and those eligible but withholding consent. Comparisons in baseline characteristics for categorical variables will be based on χ^2 test or Fisher's exact test, if expected cell counts are small. For continuous variables two-group comparisons will be performed using t-tests or the Wilcoxon rank-sum test for non-normal data. Data may not be available due to voluntary withdrawal of patients, lack of completion of individual data items or general loss to follow-up. Where possible the reasons for missing data will be ascertained and reported. If judged appropriate, missing data will be imputed using a multiple imputation strategy. Any imputation methods used for scores and other derived variables will be carefully considered and justified, and analysis of imputed datasets used to assess the sensitivity of the analysis to the missing data.

The primary analysis will be of the change in the dGEMRIC index score for a combined acetabular and femoral head cartilage ROI from baseline to 12 months, with the difference in mean change between the two treatment groups presented with a 95% confidence interval and compared using an independent samples t-test. This strategy will also be applied for the standardised dGEMRIC z-scores. Change in scores for the dGEMRIC data will be assumed to be normally distributed; possibly after appropriate variance-stabilising transformation of the individual time-point scores. Analysis including adjustment for baseline dGEMRIC index score and relevant baseline characteristics will be performed using Analysis of Covariance with the change from baseline modelled as the dependent variable. This strategy will also be applied for the dGEMRIC index score of the separate acetabular and femoral head cartilage ROIs and the standardized dGEMRIC z-scores.

The above strategy will also be performed for analysis of other approximately normally distributed secondary outcome measures including those related to plain radiography, MRI, muscle strength, full 3D gait analyses, EMG-informed NMS and FEM models, and the patient reported outcomes (iHOT-33, HOOS, EQ-5D and SF-12). Differences in dichotomous outcome variables such as adverse events, complications related to the trial interventions and the need for further procedures will be compared between groups using chi-squared tests (or Fisher's exact test). The temporal patterns of complications and the need for further procedures will be presented graphically. If there are sufficient events cumulative incidence curves of the time to first procedure (any or a specific type) will be plotted by treatment group calculated using the Kaplan-Meier method. Ordinal scores for patient satisfaction will be compared between intervention groups using proportional odds logistic regression analysis with the proportional odds assumption checked. Although our inferences will be drawn from the intention-to-treat analysis, we will perform per protocol analyses to place these in context. We plan to perform exploratory subgroup analyses by FAI type and by whether referral to the study was via the private or public healthcare systems. We do not anticipate that crossovers will be a major issue for this study. We, therefore, expect the main analyses to provide definitive results. If participant adherence to or completion of the PHT proves to be more problematic than expected, we will augment the planned analysis with a complier average causal effect (CACE) analysis.

Economic analysis

A secondary objective of this study is to assess the cumulative direct and indirect costs associated with each treatment approach and to calculate the incremental cost-effectiveness ratio per clinically meaningful change in cartilage structure (dGEMRIC). The following methods can be used to assess direct and indirect costs associated with each treatment arm. Participants will be asked for consent to researchers linking their study data to various administrative data sources (state, federal and private health insurance databases). To assess direct costs, data on healthcare utilisation over the study period (including hospital admissions, emergency presentations, primary care utilisation and pharmaceutical claims records on subsidised prescription medicines) will be extracted for all consenting participants. Health services and prescription medications will be costed using market prices for the most recent financial year [60–62].

Information on indirect costs associated with each treatment arm will be assessed at baseline, 6 and 12 months using a survey based on the modified World

Health Organisation Health and Work Performance Questionnaire (WHO HPQ). The WHO HPQ is a validated tool [38] designed to assess three domains of work performance (absenteeism, presenteeism and critical incidents), as well as basic demographic and occupational information. Participants will be asked to report their annual pre-tax income. As questions relating to income are known to have high rates of missing data, participants will also be asked to report the category of their main job (e.g. executive, service, labourer). Where annual income data are found to be missing, mean salaries for each category of employment can be imputed from the Australian Bureau of Statistics average weekly earnings data [63]. Cost estimates for lost productivity will be conducted according to previously described methods [64]. The survey will also include several questions to capture information on direct costs not obtainable through data linkage, such as outpatient physiotherapy in the public sector and non-subsidised over the counter medication utilisation.

Sample size

Sample size calculations for the primary outcome are based on statistical power of 90%, and two-sided significance of 5% significance level. With a sample size of 54 patients in each group, we expect to detect a difference of at least 50 ms between two study groups at 12 months based on a standard deviation (SD) of 80 ms for the dGEMRIC index [65, 66]. 50 ms was chosen as clinically significant based upon increased risk of subsequent total hip replacement [67].

Previous research identified an $R^2 \approx 0.2–0.3$ between indirect external measures of hip joint loading and hip neck bone density in people with hip OA, when two covariates (weight and height) were included, indicating a moderate relationship between biomechanical and structural measures. We expect higher R^2 values, given that we have direct estimates of cartilage stress and strain. Being conservative, however, in order to achieve an $R^2 > 0.2$ between cartilage loading stimulus and dGEMRIC scores (with three covariates), a power of 90%, and a two-sided significance level of 5% we require a total of 56 patients at each site. We will, therefore, aim to recruit a total of 140 participants for the study to allow for a drop-out rate of 20%, including 5% cross over to the surgical group.

Data safety and monitoring

An independent Data Safety and Monitoring Board (DSMB) will be responsible for oversight of this clinical trial, including protocol adherence, recruitment, adverse events, SAEs, treatment side effects and data analysis and data safety. The members of the DSMB are not involved in the study, do not have any conflict of interest and will not benefit in any way from the results of this trial.

Discussion

The need for RCTs to determine the most effective approach for the management of FAI is well-recognised [13]. This RCT will compare arthroscopic hip surgery to PHT for the management of FAI. Strengths of this trial include the large number of structural, biomechanical, and patient-reported outcomes by which these two treatments will be compared. Together, these outcomes are likely to provide insight into the effect of these two management approaches on the pathogenesis of hip OA, including their likely effectiveness for modification of future hip OA risk. Furthermore, the randomised, pragmatic, and multicentre nature of this trial will increase the generalisability of the results. Dissemination of results for this trial will occur through publication in peer-reviewed journals and presentation at relevant conferences.

Abbreviations

AE: Adverse events; CEINMS: Calibrated EMG-informed Neuromusculoskeletal; dGEMRIC: Delayed gadolinium-enhanced magnetic resonance imaging of cartilage; EMG-informed NMS and FEM models: Electromyography-informed neuromusculoskeletal and finite element models; EQ5D: European Quality of life-5 dimensions; FAI: Femoroacetabular impingement; GIS: Global Improvement Score; HOAMS: Hip osteoarthritis MRI score; HOOS: Hip disability and Osteoarthritis Outcome Score; iHOT-33: International Hip Outcome Tool-33; MRI: Magnetic Resonance Imaging; OA: Osteoarthritis; PHT: Personalised Hip Therapy; ROI: Region of interest; SAE: Serious adverse events; SF-12: Short-Form 12; WHO HPQ: Modified World Health Organisation Health and Work Performance Questionnaire

Funding

This study is funded by an NHMRC grant; APP1069278 and by the Australian Hip Arthroscopy Education and Research Foundation (AHAERF). Development of PHT and the protocol for UK FASHIoN were funded by UK National Institute for Health Research.

Availability of data and materials

The datasets analysed during the current study are available from the corresponding author on reasonable request.

Authors' contributions

DJH, YJK, JOD, KLB, DL, DJS, TW, JML, DG, MB, MOS, PS, StR, and ED contributed to study conception and design. JE, LS, NJM, MH, JML, MOS, JOD, PS, SuR, RM, PT, AB, CF, FMC, SMG and DJH contributed to the data collection. DJH obtained project funding. NJM and JE drafted the first version of the manuscript. JE, NJM, LS, FMC and SMG are responsible for data management. The study statistician RO will have access to the final trial dataset and perform the statistical analysis. All authors revised the protocol critically for important intellectual content and read and approved the final version of the protocol. All authors agree to be accountable for all aspects of the work.

Ethics approval and consent to participate

This protocol has been approved by St Vincent's Hospital Human Research Ethics Committee (HREC/14/SVH/343). Informed consent was obtained from all study participants before participation in the study.

Competing interests

DJH is a consultant to Flexion, TissueGene and Merck Serono and is supported by an NHMRC Practitioner Fellowship. KLB is supported by a NHMRC Principal Research Fellowship. DRG is a consultant for Stryker UK. MH is a member of the Editorial Board of BMC Musculoskeletal Disorders.

Publisher's Note

Springer Nature remains neutral with regard to jurisdictional claims in published maps and institutional affiliations.

Author details

[1]Kolling Institute of Medical Research, Institute of Bone and Joint Research, University of Sydney, Camperdown, Australia. [2]Department of Rheumatology, Royal North Shore Hospital, St Leonards, Australia. [3]Centre for Health, Exercise and Sports Medicine, Department of Physiotherapy, University of Melbourne, Melbourne, Australia. [4]Melbourne EpiCentre, University of Melbourne, Melbourne, Australia. [5]Orthopaedics ACT, 90 Corinna St., Canberra 2603, Australia. [6]Sydney Translational Imaging Laboratory, Heart Research Institute, Charles Perkins Centre, University of Sydney, Camperdown, Australia. [7]Sydney Medical School, University of Sydney, Camperdown, Australia. [8]Warwick Medical School, University of Warwick, Coventry, UK and University Hospitals of Coventry and Warwickshire NHS Trust, Coventry, UK. [9]Department of Orthopaedic Surgery, Western Health, Melbourne, Australia. [10]Australian Institute for Musculoskeletal Science (AIMSS), The University of Melbourne and Western Health, St Albans, Melbourne, VIC, Australia. [11]Department of Orthopedic Surgery, Boston Children's Hospital, 300 Longwood Avenue, Boston, MA 02115, USA. [12]Department of Musculoskeletal Imaging, Castlereagh Sports Imaging Centre, St Leonards, NSW, Australia. [13]Gold Coast Orthopaedic Research and Education Alliance, Menzies Health Institute Queensland, Griffith University, Nathan, Australia. [14]School of Allied Health Sciences, Griffith University, Nathan, Australia. [15]Sydney Orthopaedic Trauma & Reconstructive Surgery, Sydney, NSW, Australia. [16]NHMRC Clinical Trials Centre, University of Sydney, Camperdown, Australia. [17]Hip Arthroscopy Australia, 21 Erin St, Richmond, VIC, Australia. [18]St Vincent's Private Hospital, 159 Grey St, East Melbourne, VIC, Australia. [19]North Sydney Orthopaedic and Sports Medicine Centre, North Sydney, NSW, Australia. [20]Macquarie University Hospital, 3 Technology Pl, Macquarie University, Sydney, NSW 2109, Australia. [21]Institute of Social and Preventive Medicine, University of Bern, Bern, Switzerland. [22]Department of Rheumatology, Immunology and Allergology, University Hospital and University of Bern, Bern, Switzerland. [23]Maroondah Hospital, Eastern Health, Davey Drive, Ringwood East, Melbourne, VIC 3135, Australia.

Received: 15 August 2017 Accepted: 21 September 2017
Published online: 26 September 2017

References

1. Griffin DR, et al. The Warwick Agreement on Femoroacetabular Impingement. Br J Sports Med. 2016;50(19).
2. Ganz R, et al. Femoroacetabular impingement: a cause for osteoarthritis of the hip. Clin Orthop Relat Res. 2003;417:112–20.
3. Ganz R, et al. The etiology of osteoarthritis of the hip: an integrated mechanical concept. Clinical Orthopaedics & Related Research. 2008;466(2):264–72.
4. Beck M, et al. Hip morphology influences the pattern of damage to the acetabular cartilage: femoroacetabular impingement as a cause of early osteoarthritis of the hip. J Bone Joint Surg Br. 2005;87(7):1012–8.
5. Ganz R, et al. Surgical dislocation of the adult hip a technique with full access to the femoral head and acetabulum without the risk of avascular necrosis. J Bone Joint Surg Br. 2001;83(8):1119–24.
6. Ito K, et al. Femoroacetabular impingement and the cam-effect. A MRI-based quantitative anatomical study of the femoral head-neck offset. J Bone Joint Surg Br. 2001;83(2):171–6.
7. Reichenbach S, et al. Association between cam-type deformities and magnetic resonance imaging-detected structural hip damage: A cross-sectional study in young men. Arthritis Rheum. 2011;63(12):4023–30.
8. Stelzeneder D, et al. Patterns of joint damage seen on MRI in early hip osteoarthritis due to structural hip deformities. Osteoarthritis & Cartilage. 2012;20(7):661–9.
9. Sankar WN, et al. Femoroacetabular impingement: defining the condition and its role in the pathophysiology of osteoarthritis. J Am Acad Orthop Surg. 2013;21(Suppl 1):S7–s15.
10. Kowalczuk, M., et al., Does Femoroacetabular Impingement Contribute to the Development of Hip Osteoarthritis? *A Systematic Review* Sports Med Arthrosc, 2015. 23(4): p. 174–179.
11. Zeng W-N, et al. Investigation of association between hip morphology and prevalence of osteoarthritis. Sci Rep. 2016;6:23477.
12. Salaffi F, et al. Health-related quality of life in older adults with symptomatic hip and knee osteoarthritis: a comparison with matched healthy controls. Aging ClinExpRes. 2005;17(4):255–63.
13. Wall PD, et al. Surgery for treating hip impingement (femoroacetabular impingement). Cochrane Database Syst Rev. 2014;9:CD010796.
14. Wall PD, et al. Nonoperative treatment for femoroacetabular impingement: a systematic review of the literature. PM R. 2013;5(5):418–26.
15. Montgomery SR, et al. Trends and Demographics in Hip Arthroscopy in the United States. Arthroscopy. The Journal of Arthroscopic & Related Surgery. 2013;29(4):661–5.
16. Sampson TG. Arthroscopic treatment of femoroacetabular impingement. Tech Orthop. 2005;20(1):56–62.
17. Sansone, M., et al., Outcome after hip arthroscopy for femoroacetabular impingement in 289 patients with minimum 2-year follow-up. Scand J Med Sci Sports, 2016.
18. Mladenovic D, et al. Early clinical results of surgical treatment of patients with femoroacetabular impingement. Srp Arh Celok Lek. 2014;142(5–6):325–9.
19. Polesello GC, et al. Arthroscopic treatment of femoroacetabular impingement: minimum five-year follow-up. Hip International. 2014;24(4):381–6.
20. Philippon M, et al. Outcomes following hip arthroscopy for femoroacetabular impingement with associated chondrolabral dysfunction Minimum Two-Year Follow-Up. J Bone Joint Surg Br. 2009;91(1):16–23.
21. Wall PD, et al. Personalised Hip Therapy: development of a non-operative protocol to treat femoroacetabular impingement syndrome in the FASHIoN randomised controlled trial. Br J Sports Med. 2016;50(19):1217–23.
22. Enseki KR, Martin R, Kelly BT. Rehabilitation after arthroscopic decompression for femoroacetabular impingement. ClinSports Med. 2010; 29(2):247–55. viii
23. Griffin KM. Rehabilitation of the hip. Clin Sports Med. 2001;20(4):837–50. viii
24. Stalzer, S., M. Wahoff, and M. Scanlan, Rehabilitation following hip arthroscopy. Clin Sports Med, 2006. 25(2): p. 337–357, x.
25. Griffin DR, et al. Protocol for a multicentre, parallel-arm, 12-month, randomised, controlled trial of arthroscopic surgery versus conservative care for femoroacetabular impingement syndrome (FASHIoN). BMJ Open. 2016; 6(8):e012453.
26. Griffin D, et al. UK FASHIoN: feasibility study of a randomised controlled trial of arthroscopic surgery for hip impingement compared with best conservative care. Health Technol Assess. 2016;20(32):1–172.
27. Griffin DR, et al. The feasibility of conducting a randomised controlled trial comparing arthroscopic hip surgery to conservative care for patients with femoroacetabular impingement syndrome: the FASHIoN feasibility study. Journal of Hip Preservation Surgery. 2016;3(4):304–11.
28. Nötzli H, et al. The contour of the femoral head-neck junction as a predictor for the risk of anterior impingement. Journal of Bone & Joint Surgery, British Volume. 2002;84(4):556–60.
29. Nepple JJ, et al. Diagnostic imaging of femoroacetabular impingement. J Am Acad Orthop Surg. 2013;21(suppl):S20–6.
30. Tönnis D, Heinecke A. Current Concepts Review-Acetabular and Femoral Anteversion: Relationship with Osteoarthritis of the Hip*. The Journal of Bone & Joint Surgery. 1999;81(12):1747–70.
31. Lattanzi R, et al. A new method to analyze dGEMRIC measurements in femoroacetabular impingement: preliminary validation against arthroscopic findings. Osteoarthritis & Cartilage. 2012;20(10):1127–33.
32. Lattanzi R, et al. Detection of cartilage damage in femoroacetabular impingement with standardized dGEMRIC at 3 T. Osteoarthritis & Cartilage. 2014;22(3):447–56.
33. Zilkens C, et al. Current knowledge and importance of dGEMRIC techniques in diagnosis of hip joint diseases. Skelet Radiol. 2015;44(8):1073–83.
34. Mamisch TC, et al. Delayed gadolinium-enhanced magnetic resonance imaging of cartilage (dGEMRIC) in Femoacetabular impingement. J Orthop Res. 2011;29(9):1305–11.
35. Domayer SE, et al. Radial dGEMRIC in developmental dysplasia of the hip and in femoroacetabular impingement: preliminary results. Osteoarthritis & Cartilage. 2010;18(11):1421–8.
36. Bittersohl B, et al. Reproducibility of dGEMRIC in assessment of hip joint cartilage: a prospective study. J Magn Reson Imaging. 2009;30(1):224–8.
37. Bashir A, et al. Nondestructive imaging of human cartilage glycosaminoglycan concentration by MRI. Magn Reson Med. 1999;41(5):857–65.
38. Kessler RC, et al. The World Health Organization. Health and Work Performance Questionnaire (HPQ). J Occup Environ Med. 2003;45(2): 156–174.
39. Fransen M, McConnell S. Exercise for osteoarthritis of the knee. Cochrane Database Syst Rev. 2008;4:CD004376.
40. *HOOS* User's Guide. 2003; Available from: http://www.koos.nu/HOOSEng.pdf.
41. Kemp JL, et al. Psychometric properties of patient-reported outcome measures for hip arthroscopic surgery. Am J Sports Med. 2013;41(9):2065–73.

42. Hinman RS, et al. Which is the most useful patient-reported outcome in femoroacetabular impingement? Test-retest reliability of six questionnaires. Br J Sports Med. 2014;48(6):458–63.
43. Walters SJ, Brazier JE. Comparison of the minimally important difference for two health state utility measures: EQ-5D and SF-6D. Qual Life Res. 2005; 14(6):1523–32.
44. Ostendorf M, et al. Patient-reported outcome in total hip replacement. A comparison of five instruments of health status. J Bone Joint Surg Br. 2004; 86(6):801–8.
45. Roemer FW, et al. Hip Osteoarthritis MRI Scoring System (HOAMS): reliability and associations with radiographic and clinical findings. Osteoarthr Cartil. 2011;19(8):946–62.
46. Tomczak RJ, et al. MR imaging measurement of the femoral antetorsional angle as a new technique: comparison with CT in children and adults. Am J Roentgenol. 1997;168(3):791–4.
47. Hesper T, et al. Both 3-T dGEMRIC and Acetabular-Femoral T2 Difference May Detect Cartilage Damage at the Chondrolabral Junction. Clin Orthop Relat Res. 2017;475(4):1058–65.
48. Rosset A, Spadola L, Ratib O. OsiriX: An Open-Source Software for Navigating in Multidimensional DICOM Images. J Digit Imaging. 2004;17(3): 205–16.
49. Bittersohl B, et al. Cartilage damage in femoroacetabular impingement (FAI): preliminary results on comparison of standard diagnostic vs delayed gadolinium-enhanced magnetic resonance imaging of cartilage (dGEMRIC). Osteoarthritis & Cartilage. 2009;17(10):1297–306.
50. Schmaranzer F, et al. How Does the dGEMRIC Index Change After Surgical Treatment for FAI? A Prospective Controlled Study: Preliminary Results. Clin Orthop Relat Res. 2017;475(4):1080–99.
51. Hingsammer AM, et al. Does periacetabular osteotomy for hip dysplasia modulate cartilage biochemistry? J Bone Joint Surg (Am Vol). 2015;97(7): 544–50.
52. Clohisy JC, et al. A systematic approach to the plain radiographic evaluation of the young adult hip. J Bone Joint Surg Am. 2008;90(Suppl 4):47–66.
53. Tannast M, et al. Radiographic analysis of femoroacetabular impingement with Hip2Norm-reliable and validated. JOrthopRes. 2008; 26(9):1199–205.
54. Saxby DJ, et al. Tibiofemoral Contact Forces in the Anterior Cruciate Ligament-Reconstructed Knee. Med Sci Sports Exerc. 2016;48(11):2195–206.
55. Hermens HJ, et al. Development of recommendations for SEMG sensors and sensor placement procedures. J Electromyogr Kinesiol. 2000;10(5):361–74.
56. Pizzolato C, et al. CEINMS: A toolbox to investigate the influence of different neural control solutions on the prediction of muscle excitation and joint moments during dynamic motor tasks. J Biomech. 2015;48(14):3929–36.
57. Fernandez JM, et al. Bone remodelling in the natural acetabulum is influenced by muscle force induced bone stress. International Journal for Numerical Methods in Biomedical Engineering. 2014;30(1):28–41.
58. Zhang J, et al. The MAP Client: User-Friendly Musculoskeletal Modelling Workflows Biomedical Simulation, Lecture Notes in Computer Science, ed. F. Bello and S. Cotin. Vol. 8789. 2014, Switzerland: Springer International.
59. Maas SA, et al. FEBio: finite elements for biomechanics. J Biomech Eng. 2012;134(1):011005.
60. Felson D, et al. The indidence and natural history of knee osteoarthritis in the elderly. The Framingham Osteoarthritis Study. Arthritis Rheum. 1995;38:1500–5.
61. Gudbergsen H, et al. Weight loss is effective for symptomatic relief in obese subjects with knee osteoarthritis independently of joint damage severity assessed by high-field MRI and radiography. Osteoarthritis & Cartilage. 2012; 20(6):495–502.
62. Australian Government of Health and Ageing. National Hospital Cost Data Collection Round 16 (2011–2012). 2013.
63. Australian Bureau of Statistics. Average Weekly Earnings, Australia, May 2013. 2013. Canberra: Australian Bureau of Statistics.
64. Kessler RC, et al. Using the World Health Organization Health and Work Performance Questionnaire (HPQ) to Evaluate the Indirect Workplace Costs of Illness. J Occup Environ Med. 2004. 46(Supplement):S23-S37.
65. Domayer SE, et al. Radial dGEMRIC in developmental dysplasia of the hip and in femoroacetabular impingement: preliminary results. Osteoarthr Cartil. 2010;18(11):1421–8.
66. Kim YJ, et al. Assessment of early osteoarthritis in hip dysplasia with delayed gadolinium-enhanced magnetic resonance imaging of cartilage. J Bone Joint Surg (Am Vol). 2003;85-A(10):1987–92.
67. Cunningham T, et al. Delayed gadolinium-enhanced magnetic resonance imaging of cartilage to predict early failure of Bernese periacetabular osteotomy for hip dysplasia. J Bone Joint Surg Am. 2006. 88(7):1540–1548.

Submit your next manuscript to BioMed Central and we will help you at every step:

- We accept pre-submission inquiries
- Our selector tool helps you to find the most relevant journal
- We provide round the clock customer support
- Convenient online submission
- Thorough peer review
- Inclusion in PubMed and all major indexing services
- Maximum visibility for your research

Submit your manuscript at
www.biomedcentral.com/submit

BioMed Central
